# Triazine-Based Porous Organic Polymers: Synthesis and Application in Dye Adsorption and Catalysis

**DOI:** 10.3390/polym15081815

**Published:** 2023-04-07

**Authors:** Pedro M. C. Matias, Dina Murtinho, Artur J. M. Valente

**Affiliations:** Department of Chemistry, CQC-IMS, University of Coimbra, Rua Larga, 3004-535 Coimbra, Portugal; petermatias1998@gmail.com (P.M.C.M.); dmurtinho@ci.uc.pt (D.M.)

**Keywords:** porous organic polymers, triazine, dye adsorption, heterogeneous catalysis

## Abstract

The scientific community has been developing promising materials to increase the sustainability and efficiency of production processes and pollutant environmental remediation strategies. Porous organic polymers (POPs) are of special interest, as they are insoluble custom-built materials at the molecular level, endowed with low densities and high stability, surface areas, and porosity. This paper describes the synthesis, characterization, and performance of three triazine-based POPs (T-POPs) in dye adsorption and Henry reaction catalysis. T-POPs were prepared by a polycondensation reaction between melamine and a dialdehyde (terephthalaldehyde (T-POP1) or isophthalaldehyde derivatives with a hydroxyl group (T-POP2) or both a hydroxyl and a carboxyl group (T-POP3)). The crosslinked and mesoporous polyaminal structures, with surface areas between 139.2 and 287.4 m^2^ g^−1^, positive charge, and high thermal stability, proved to be excellent methyl orange adsorbents, removing the anionic dye with an efficiency >99% in just 15–20 min. The POPs were also effective for methylene blue cationic dye removal from water, reaching efficiencies up to ca. 99.4%, possibly due to favorable interactions via deprotonation of T-POP3 carboxyl groups. The modification of the most basic polymers, T-POP1 and T-POP2, with copper(II) allowed the best efficiencies in Henry reactions catalysis, leading to excellent conversions (97%) and selectivities (99.9%).

## 1. Introduction

Nanoporous materials (NMs), which include zeolites, metal–organic frameworks (MOFs), and porous organic frameworks (POFs), have found remarkable scientific progress in recent decades due to their excellent performance in diverse domains [1,2,3,4,5,6].

POFs are a class of polymers made up of organic building units of non-metallic elements linked by strong covalent bonds. In terms of structural regularity, POFs can be classified as amorphous porous organic polymers (POPs) or crystalline organic frameworks (COFs). POPs are the major class of POFs, comprising some subclasses, such as hypercrosslinked polymers (HCPs), polymers of intrinsic microporosity (PIMs), conjugated microporous polymers (CMPs), and porous aromatic frameworks (PAFs). POFs show some advantages when compared to other nanoporous materials, such as zeolites and MOFs, including higher surface areas, easier design and modification, and higher stability under severe conditions [4]. Additionally, its low density, high physicochemical stability, and surface area, together with a significant density of active sites and improved control over composition, topology, and porosity, make POFs ideal candidates for heterogeneous catalysis [7,8,9,10], important for the development of more sustainable production processes, and for water and soil remediation [11,12].

In general, the types of active sites determine the catalytic properties of a material. Catalytic POFs can be built through a bottom-up (or pre-synthetic) approach, directly using monomers with active catalytic sites, giving rise to POFs with a high density of those interfacial sites, uniformly distributed into the structure; since POFs are organic compounds and show the ease of introduction of other molecular catalysts in its structure, they can be modulated by a post-synthetic strategy, in order to optimize its performance in catalysis, obtaining materials with activities and selectivities comparable to homogeneous counterparts. Furthermore, the polymeric essence of POFs gives high catalyst stability and facilitates its handling, recovery, and reuse, which are characteristics of heterogeneous systems, and which can increase its catalytic performance [13,14,15]. Thus, the use of POFs as catalysts is of high relevance as it combines the advantages of homogeneous and heterogeneous catalysis [16].

Much of the properties that allow a given material to be used as a catalyst are also important for its potential as a good performance adsorbent. Nowadays, the development of nanoporous materials for water remediation has been of paramount interest [17,18]. Among different techniques to do so, adsorption processes are one of the most advantageous, and are used in water purification technologies because of their durability, efficiency, applicability to various pollutants, sustainability, economical operation design, selectivity, and/or promising capture capacity [19,20]. As the extent of adsorption is determined by the surface area of the adsorbents, it is essential to opt for the use of highly porous small particles [20], being POFs promising candidates as porous adsorbent materials for the removal and adsorption of contaminants from water [12,21,22,23].

The existence of easily scalable and economical synthetic strategies and the high robustness of these polymers is easily combined with their structural versatility, which allows an adjustment of the material surface concerning the following: (i) the pore size for improving the diffusion of analytes, the accommodation of a specific pollutant, and the rate of removal; (ii) the introduction of functional groups to increase the density of strong adsorption sites and its cooperation, maintaining the robustness of the structural backbone by providing specific interactions with a given reactant/pollutant; and (iii) the surface area, typically elevated [24].

Recent studies show the existence of different materials, including POFs, for heterogeneous catalysis of different reactions [9] or for the removal of contaminants from water [12]. Due to the importance of developing multipurpose structures, the novelty of this work is based on the exploration of low-cost dual-purpose POPs, both in catalysis and in adsorption. In this context, this work describes three amorphous [25] triazine-based POPs with aminal structures (T-POPs) that were synthesized by a polycondensation reaction between melamine (a triamine—$C_{3}$ geometry) and $C_{2}$ functionalized and non-functionalized dialdehydes. The presence of different functionalities in the aldehyde moiety allowed us to determine how different properties (e.g., pore size, surface area, surface charge, and wall modification) affect the performance of the T-POPs in dye (methyl orange and methylene blue) adsorption and in the catalysis of Henry reactions, in which the catalytic activity of polymers directly used as organocatalyst was compared with the performance of its Cu(II)-modified derivatives.

## 2. Materials and Methods

### 2.1. Materials

The following materials and solvents were used: melamine (99%), terephthalaldehyde (99%), 4-ethylphenol (97%), 4-hydroxybenzoic acid (99%), 2-nitrobenzaldehyde (98%), 3-nitrobenzaldehyde (99%), 2-chlorobenzaldehyde (99%), 2-methylbenzaldehyde (97%), 2-bromobenzaldehyde (98%), 4-methylbenzaldehyde (97%), 4-bromobenzaldehyde (99%), 1-naphthaldehyde (95%), and 4-carboxybenzaldehyde (97%) were purchased from Sigma-Aldrich (Schnelldorf, Germany); glacial acetic acid (≥99%), potassium hydrogen phthalate (≥99.9%), and methyl orange were supplied by Merck (Darmstadt, Germany); hexamethylenetetramine (≥99%), nitromethane (99%), and methanol (≥99.8%) were purchased from Riedel-de Haën (Seelze, Germany); standard solution 1000 mg L^−1^ Cu(II) in 0.5 M nitric acid and methylene blue were purchased from PanReac (Barcelona, Spain); anhydrous sodium sulphate (99.7%), sodium chloride (>99%), sulfuric acid (96%), acetone (>99.6%), and sodium hydroxide (99.4%) were acquired from José Manuel Gomes dos Santos (Lisboa, Portugal); nitric acid (65%) and hydrochloric acid (37%) were purchased from Chem-Lab (Zedelgem, Belgium); 3-chlorobenzaldehyde, 3-methylbenzaldehyde, 2-naphthaldehyde were provided by Fluorochem (Hadfield, UK); tetrahydrofuran (≥99.9%) and cyclohexane (≥99.8%) were received from Carlo Erba (Val de Reuil, France); and deuterated chloroform (CDCl_3_, 99.8%D) and dimethylsulfoxide (DMSO-d_6_, 99.8%D) were purchased from Eurisotop (Saint-Aubin, France). Other reagents and solvents were used: 4-nitrobenzaldehyde (99%, Alfa Aesar, Karlsruhe, Germany), benzaldehyde (98%, Thermo Fisher Scientific, Geel, Belgium), trifluoroacetic acid (99.9%, Fluorochem, Hadfield, UK), dimethylsulfoxide (≥99.5%, Riedel-de Haën, Seelze, Germany), and copper(II) acetate monohydrate (≥98%, J.T. Baker, Center Vally, PA, USA).

All materials and solvents, with the exception of terephthalaldehyde, were used as received.

### 2.2. Synthesis of the Porous Organic Polymers (T-POPs)

The porous organic polymers with aminal structure and based on triazine rings (T-POPs) were obtained by reaction of melamine with a dialdehyde. Among the dialdehydes, a commercially available non-functionalized derivative (terephthalaldehyde) and functionalized monomers (4-ethyl-2,6-diformylphenol (EDP) and 3,5-diformyl-4-hydroxybenzoic acid (DHA)) were used for obtaining T-POP1, T-POP2, and T-POP3, respectively.

(1) Terephthalaldehyde: High-purity terephthalaldehyde was obtained by recrystallization using the method described by Yoon et al. [26]. Briefly, terephthalaldehyde (5.00 g) was dissolved in methanol (40 mL) under stirring and slight heating, and the undissolved acid fraction was filtered. Then, water (200 mL) was added to precipitate the aldehyde, which was filtered off and vacuum dried. ^1^H-NMR (400 MHz, CDCl_3_, ppm): 8.05 (s, 4H, H–Ar); 10.13 (s, 2H, CHO). IR (cm^−1^): 769, 813, 1009, 1090, 1098, 1196, 1299, 1334, 1367, 1385, 1431, 1498, 1687, 2758, 2806, 2865.

The functionalized monomers were prepared by diformylation of phenol derivatives through Duff reaction:

(2) 4-ethyl-2,6-diformylphenol (EDP): 4-ethylphenol (4.18 g, 34.2 mmol) and hexamethylenetetramine (9.60 g, 68.5 mmol) were dissolved in trifluoroacetic acid (60 mL). The resulting solution was refluxed (100 °C) with magnetic stirring and under an inert atmosphere for 24 h. After this time, the cooled reaction mixture was poured into an Erlenmeyer flask containing hydrochloric acid 4 M (50 mL) and stirred for 10 min, and then it was extracted using dichloromethane (2 × 150 mL). The combined organic phases were extracted with hydrochloric acid 4 M (2 × 100 mL), distilled water (200 mL), and finally, a saturated solution of sodium chloride (200 mL). The final organic fraction was dried with anhydrous sodium sulfate, filtered under reduced pressure, and evaporated, yielding a yellow-brown solid residue [27]. The pure product was collected as a fine yellow powder, after recrystallization with cyclohexane, in 21% yield. ^1^H-NMR (400 MHz, CDCl_3_, ppm): 1.27 (t, 3H, CH_3_, J = 7.6 Hz); 2.69 (q, 2H, CH_2_, J = 7.6 Hz); 7.80 (s, 2H, H–Ar); 10.23 (s, 2H, CHO); 11.47 (s, 1H, OH). IR (cm^−1^): 744, 789, 908, 972, 1003, 1066, 1154, 1201, 1267, 1298, 1326, 1377, 1403, 1444, 1456, 1596, 1661, 1679, 2777, 2868, 2932, 2967, 3142.

(3) 3,5-diformyl-4-hydroxybenzoic acid (DHA): 4-hydroxybenzoic acid (1.10 g, 8.0 mmol) and hexamethylenetetramine (8.97 g, 64.0 mmol) were dissolved in trifluoroacetic acid (40 mL), and the reaction mixture was maintained at reflux (110 °C) with magnetic stirring and under an inert atmosphere for 72 h. After cooling, the solution was poured into an Erlenmeyer flask containing hydrochloric acid 4 M (200 mL) and stirred vigorously for 30 min. Thereafter, the mixture was allowed to stand for 72 h to allow a precipitate to form [28]. The solid product formed was filtered under reduced pressure, washed with distilled water (3 × 20 mL), and dried in vacuo. A pure yellow solid was obtained with a yield of 66%. ^1^H-NMR (400 MHz, DMSO-d_6_, ppm): 8.55 (s, 2H, H–Ar); 10.30 (s, 2H, CHO). IR (cm^−1^): 727, 771, 796, 810, 931, 942, 1002, 1124, 1166, 1204, 1264, 1295, 1348, 1365, 1388, 1587, 1647, 1685, 1717, 2765, 2874, 3040, 3063, 3308.

The synthesis of porous polymers T-POP1, T-POP2, and T-POP3 was carried out by reaction between melamine (0.3847 g; 3.05 mmol) and the respective dialdehyde (4.58 mmol): terephthalaldehyde, 4-ethyl-2,6-diformylphenol (EDP), or 3,5-diformyl-4-hydroxybenzoic acid (DHA), dissolved in 20 mL of DMSO and 7.50 mL of 3 M acetic acid [29]. A reflux condenser was attached to the round bottom flask, and the mixture was heated to 140 °C, in an inert atmosphere and under magnetic stirring for 48 h (T-POP1 and T-POP3) or 72 h (T-POP2). In each case, the formed precipitate was filtered under gravity, washed with THF, acetone, and methanol, and dried in an oven at 40 °C. The final products were obtained in the form of fine powders of white (T-POP1), beige (T-POP2), and yellow (T-POP3) colors, in 61%, 68%, and 53% yield, respectively.

IR_T-POP1_ (cm^−1^): 669, 747, 813, 875, 986, 1016, 1103, 1153, 1189, 1342, 1475, 1542, 2960, 3391.

IR_T-POP2_ (cm^−1^): 669, 745, 813, 875, 952, 988, 1016, 1113, 1153, 1192, 1342, 1475, 1542, 2960, 3384.

IR_T-POP3_ (cm^−1^): 672, 750, 813, 876, 988, 1014, 1107, 1156, 1196, 1342, 1475, 1542, 1653, 2960, 3393.

For catalytic purposes, T-POP1 or T-POP2 (200 mg) were metalated using a solution of Cu(OAc)_2_∙H_2_O (159.7 mg; 0.80 mmol) in 60 mL of methanol, and then the suspension was heated at 60 °C under magnetic stirring for 24 h. After reaction, the metalated polymer (Cu@T-POP) was filtered under reduced pressure, washed with methanol and acetone, and dried in an oven at 40 °C. This strategy allowed a retention of Cu(II) equal to (217 ± 10) and (178 ± 10) mg g^−1^ for T-POP1 and T-POP2, respectively, corresponding to (682 ± 30) and (559 ± 32) mg g^−1^ in Cu(OAc)_2_∙H_2_O and adsorption efficiencies of (85 ± 4)% and (70 ± 4)%.

### 2.3. Characterisation of Monomers and POPs

(1) Infrared spectroscopy: Attenuated total reflectance Fourier transform infrared spectra (FTIR-ATR) were recorded at room temperature, in the 4000–650 cm^−1^ wavenumber range, using an Agilent Technologies Cary 630 FTIR spectrometer.

(2) Thermogravimetric Analysis (TGA): Thermogravimetric analysis was carried out by using a Nietzsch Tarsus TG 209 F3 analyzer, in a 25–700 °C temperature range, at a heating rate of 10 °C min^−1^ and a nitrogen purge flow rate of 50 mL min^−1^.

(3) Scanning electron microscopy (SEM): For an evaluation of the surface morphology, SEM micrographs were taken at 1 or 2 kV by using a field emission scanning electron microscope FE-SEM Zeiss Merlin Gemini 2. Before that, samples were frozen in liquid nitrogen, freeze-dried for 24 h on a Labconco Freezone 4.5 device, and coated with a thin gold film.

(4) Surface area and porosimetry: The nitrogen adsorption–desorption isotherms of polymers were performed in a Micromeritics ASAP 2000 apparatus to determine the specific surface area (*S*_BET_), the specific volume of pores (*V*_p_) and the average size of each pore (*d*_p_). The pore size was computed by using the Brunauer–Emmett–Teller (BET) model through the following equation: ($\frac{4V_{p}}{S_{BET}}$).

(5) Dynamic and electrophoretic light scattering: Dynamic light scattering (DLS) and ζ-potential measurements were performed by using a Malvern Zetasizer NanoZS; the measurements were carried out by using 1 mL of polymer dispersion in milli-Q water at 25 °C.

(6) Potentiometry: For the assessment of carboxyl groups content in the T-POP3, potentiometric titration was carried out using the following experimental conditions: 15.2 mg of T-POP3 was suspended in 25 mL of water and titrated with a 0.584 mM NaOH solution, previously standardized with a 5 mM potassium hydrogen phthalate solution. A pH meter from Radiometer Copenhagen MeterLab PHM240 (Radiometer, Copenhagen, Denmark), coupled with an Ingold U457-K7pH conjugated electrode, was used.

(7) Flame atomization atomic absorption spectroscopy (F-AAS): Cu@T-POP1 and Cu@T-POP2 (20.0 mg) were subjected to digestion in 65% nitric acid (5 mL), refluxing the mixture at 90 °C until it becomes translucent (4 h). After appropriate dilution of the resulting solutions, Cu(II) concentration was quantified by using an atomic absorption spectrometer Unicam Solaar 939, equipped with a copper hollow cathode lamp (λ = 325 nm; slit = 37 mm) and an air/acetylene flame (optical path~10 cm).

(8) Nuclear magnetic resonance spectroscopy: Proton nuclear magnetic resonance (^1^H-NMR) spectra were performed on a Bruker Avance III spectrometer, 400 MHz. CDCl_3_ or DMSO-d_6_ were used as deuterated solvents and tetramethylsilane (TMS) as internal standard, with chemical shifts expressed in ppm and coupling constants (*J*) in Hz.

### 2.4. Adsorption Isotherms and Kinetics

The sorption analyses for polymers were performed in aqueous solution of dye, shaken at 120 rpm and at 25 °C, using a solid–liquid ratio (R_S-L_) of 2 mg mL^−1^. All experiments were carried out in duplicate, and dye quantification was performed by UV-vis spectroscopy (Shimadzu UV-2600i), using quartz cuvette with a 1 cm optical path, from the maximum absorbance at 463 nm for methyl orange (MO) and 663 nm for methylene blue (MB). The amount of dye adsorbed per unit mass of adsorbent at equilibrium ($q_{e}$, mg g^−1^) and the removal efficiency (*Q*(%)) was assessed using Equations (1) and (2), where $C_{0}$ and $C_{e}$ (mg L^−1^) are the initial and equilibrium dye concentrations, respectively, V (L) is the volume of solution, and *m* (g) is the mass of adsorbent.

(1) $$q_{e}=\frac{\left(C_{0}-C_{e}\right)V}{m}$$

(2) $$Q(\%)=\frac{C_{0}-C_{e}}{C_{0}}\times 100$$

Sorption isotherms were obtained in the dye concentration range of 0–800 mg L^−1^ and after an equilibrium time equal to 24 h. Different models were investigated to fit the experimental data, namely the Langmuir (Equation (3)), Freundlich (Equation (4)), and Hill (Equation (5)) equations:

(3) $$q_{e}=\frac{q_{m}K_{L}C_{e}}{1+K_{L}C_{e}}$$

(4) $$q_{e}={K_{F}C_{e}}^{1/n_{F}}$$

(5) $$q_{e}=\frac{q_{S_{H}}C_{e}^{n_{H}}}{K_{D}+C_{e}^{n_{H}}}$$

where $q_{m}$ (mg g^−1^) and $K_{L}$ (L mg^−1^) define, respectively, the maximum adsorption capacity and the equilibrium constant, both given by Langmuir model [30]; $K_{F}$ (${mg}^{1-1/n_{F}}g^{-1}L^{1/n_{F}}$) and ($1/n_{F}$) are the Freundlich constant and the surface heterogeneity factor [31], and $q_{S_{H}}$ (mg g^−1^), $n_{H}$ and $K_{D}$ ((mg L^−1^)${)}^{n_{H}})$ are the maximum adsorption capacity calculated by the Hill isotherm, the Hill cooperativity coefficient, and the Hill constant, respectively [32,33].

Sorption kinetics were performed using dye aqueous solution of 10 mg L^−1^, and the pseudo-first (Equation (6)) and second (Equation (7)) order kinetic equations were used to evaluate the mechanism [31,34,35]:

(6) $$q_{t}={q_{e}(1-e}^{-k_{1}t})$$

(7) $$q_{t}=\frac{k_{2}q_{e}^{2}t}{1+k_{2}q_{e}t}$$

where $q_{t}$ (mg g^−1^) is the amount of analyte adsorbed at time *t* (min), and $k_{1}$ (min^−1^) and $k_{2}$ (g mg^−1^ min^−1^) are the pseudo-first-order and pseudo-second-order rate constants, respectively.

The goodness of different fit models was evaluated through the coefficient of determination (R^2^) and the Akaike information criterion (AIC), Equation (8) [36].

(8) $$AIC=n\log\left(\frac{s^{2}}{n}\right)+2K$$

where $s^{2}$ is the residual sum of squares, n is the number of experimental data points, and K is the number of model parameters.

### 2.5. General Procedure for Henry Reactions

In general, the aldehyde substrate (0.80 mmol), an excess of nitromethane (2 mL), used as solvent and reagent, and the synthesized catalysts (20.0 mg) were added in a glass vial. Then, the reaction mixture was stirred for 48 h at 40 °C or 60 °C. A blank reaction (without catalyst) was made at 60 °C using 4-nitrobenzaldehyde as substrate, and a control reaction was performed using 4-nitrobenzaldehyde, and Cu(OAc)_2_∙H_2_O (8.3 mg) as catalyst.

After the reaction, the catalyst (when used) was recovered by filtration, followed by washing with dichloromethane. The solution was evaporated under reduced pressure, and the residue was dissolved in CDCl_3_ and analyzed by ^1^H-NMR spectroscopy to quantify the reaction conversion (Equation (9)) and 2-nitroalcohol selectivity (Equation (10)). The peaks areas corresponding to the CHO resonance (A_CHO_), the ^1^H resonance of the hydrogen linked to the carbon containing the hydroxyl group of the 2-nitroalcohol (A_CH_), and the proton with the highest chemical shift of the R=C–H function of the nitroalkene (A_R=C–H_), were used to calculate the conversions and selectivities.

(9) $$Conversion(\%)=\frac{A_{CH}+A_{R=C-H}}{A_{CHO}+A_{CH}+A_{R=C-H}}\times 100$$

(10) $$Selectivity(\%)=\frac{A_{CH}}{A_{CH}+A_{R=C-H}}\times 100$$

The reuse cycles of heterogeneous catalysts (Cu@T-POP1 and Cu@T-POP2) were carried out under the optimized conditions for the Henry reaction between 4-nitrobenzaldehyde and nitromethane, i.e., conventional heating at 40 °C for 48 h. 4-nitrobenzaldehyde was selected as the aldehyde substrate, given the best results obtained using this compound. For each catalyst, 5–6 reuse cycles were performed. After each reaction, the mixture was subjected to centrifugation for 10 min at 8000 rpm, followed by liquid-phase decantation. The remaining solid (catalyst) was washed three times using dichloromethane and subjected to centrifugation and decantation each time. The remaining catalyst was then dried in an oven at 40 °C, before the addition of a new reactant fraction (4-nitrobenzaldehyde and nitromethane) to carry out a new catalytic cycle. The percentages of conversion and selectivity in each recycling step were determined as above.

## 3. Results and Discussion

### 3.1. Synthesis and Characterisation of T-POPs

#### 3.1.1. Synthesis of Monomers and T-POPs

**Figure 1:**
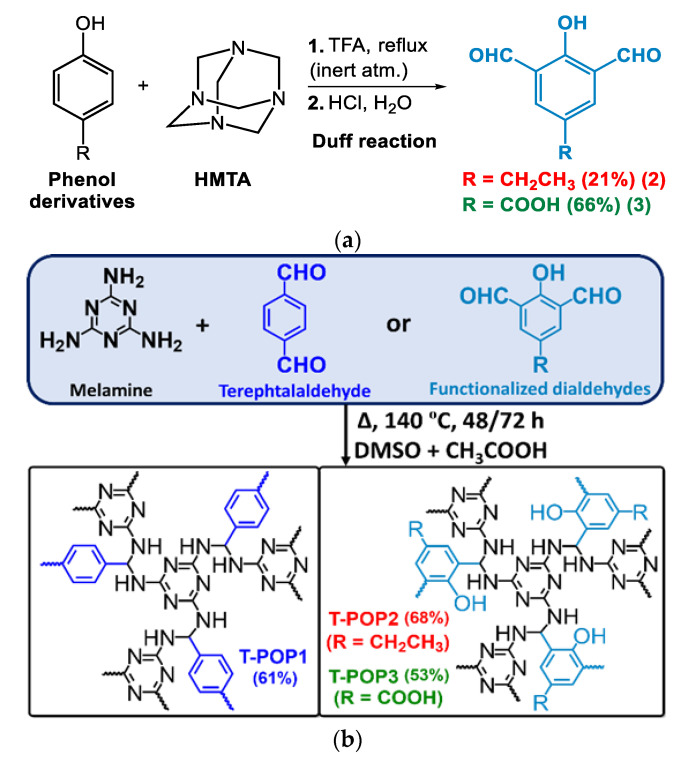
(**a**) Duff reaction to obtain functionalized dialdehydes; (**b**) polycondensation reaction between melamine and a dialdehyde to obtain T-POPs.

Three POPs were synthesized by a polycondensation reaction between melamine and a dialdehyde in a molar ratio of 2:3 [29]. Terephthalaldehyde, 4-ethyl-2,6-diformylphenol (EDP), and 3,5-diformyl-4-hydroxybenzoic acid (DHA) were chosen as aldehydes, and the respective polymers, named T-POP1, T-POP2, and T-POP3 (Figure 1), were obtained in mass yields of 61, 68, and 53%. Among the dialdehydes, the functionalized ones, EDP and DHA, were previously prepared by diformylation of the phenolic precursors (4-ethylphenol or 4-hydroxybenzoic acid) in the ortho positions relative to the activating hydroxyl group, via Duff reaction (Figure 1a) [27,28]. Polymer formation occurs through the reaction between melamine and the aldehyde, forming an imine, followed by a nucleophilic attack of the amine group of a second melamine molecule to the imine bond to form substituted secondary amines (–NH–C(R)–NH–) (Figure 1b) [37,38].

For catalytic essays, T-POP1 and T-POP2 were also metalated in a methanolic copper(II) acetate solution at 60 °C to obtain the respective Cu@T-POP1 and Cu@T-POP2 derivatives.

#### 3.1.2. Infrared Spectroscopy (FTIR-ATR)

**Figure 2:**
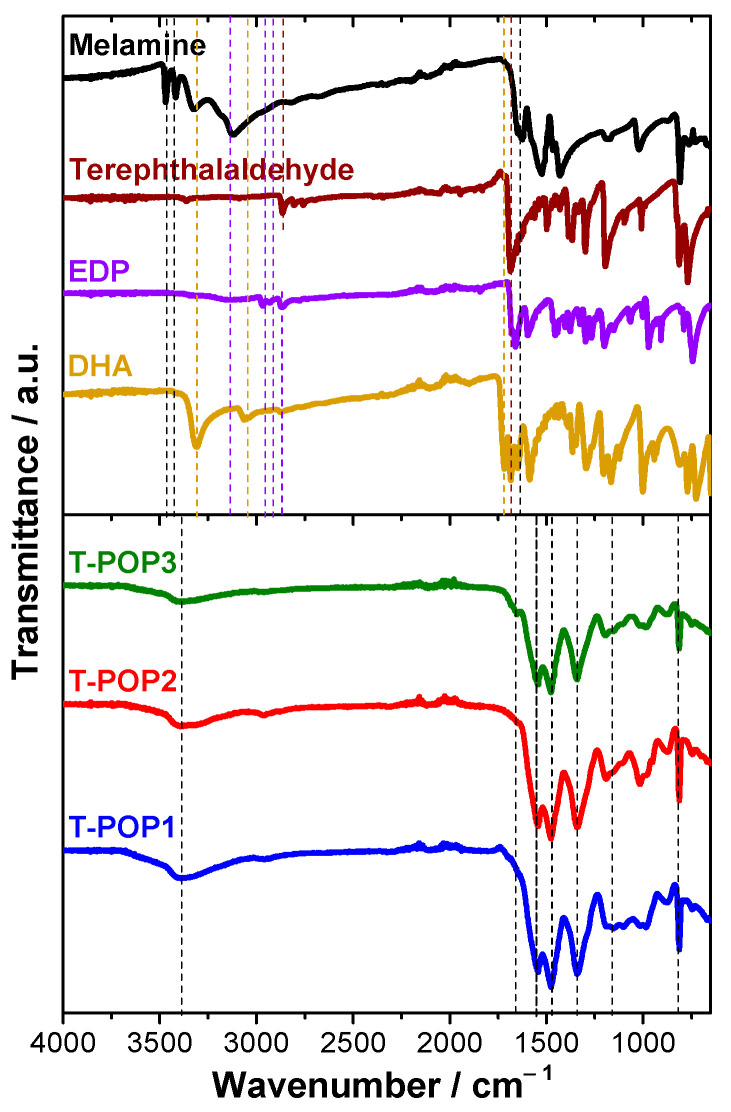
FTIR spectra of monomers (melamine, terephthalaldehyde, 4-ethyl-2,6-diformylphenol (EDP), 3,5-diformyl-4-hydroxybenzoic acid (DHA)) and polymers (T-POP1, T-POP2, T-POP3).

The information about chemical interactions and functional groups of monomers and polymers was assessed by FTIR-ATR (Figure 2). Dialdehydes present an intense band at 1661–1687 cm^−1^ corresponding to the elongation of the C=O bond and a C–H elongation band at 2865–2874 cm^−1^, both characteristic of formyl functions. Primary amine groups of melamine showed N–H elongation bands at 3420 cm^−1^ and 3470 cm^−1^, and a N–H deformation band at 1650 cm^−1^. After the polycondensation reaction between melamine and a dialdehyde, the three structurally similar polymers (T-POPs) showed no evidence of C=O and C–H elongations characteristics of formyl groups, nor vibration bands of NH_2_ groups of melamine, nor C=N elongation peaks of imine bonds (1600 cm^−1^). On the other hand, vibrational modes typical of the formation of secondary amine groups appear on polymers, namely C–N bending (813 cm^−1^), C–N elongations of (–HN–C(R)–NH–) function (1153 cm^−1^), C–NAr elongations of secondary arylamine moieties (1342 cm^−1^), and N–H elongation band (centered at 3384–3393 cm^−1^), confirming their polyaminal structure. At 1542 cm^−1^ and 1475 cm^−1^ are also observed C=N vibration bands characteristic of the heteroaromatic triazine rings incorporated into the polymeric backbones [25,37,39,40,41].

Despite formyl group vibration bands, EDP for T-POP2 synthesis also presents peaks due to C–H asymmetric elongation of methylene and methyl groups of the ethyl substituent, at 2932 cm^−1^ and 2967 cm^−1^ [41], respectively, and due to O–H elongation of the hydroxyl group at 3142 cm^−1^, which appears as a weak and broad band, as a result of the intramolecular hydrogen bonds formed with the formyl groups in ortho position [42]. Subsequently, in T-POP2, the C–H elongation vibrations of the ethyl groups appear as a wider absorption band at 2960 cm^−1^, and the O–H elongations are energetically superimposed with N–H ones, in a broad peak (~3400 cm^−1^) resulting from the involvement in hydrogen bonds. The functionalized dialdehyde DHA also shows the O–H elongations of the hydroxyl group, at 3040–3064 cm^−1^, with the same characteristics as for EDP, as well as an intense and narrow band of C=O elongations at 1717 cm^−1^, and a broad and intense band of O–H elongations at 3308 cm^−1^, both attributable to the carboxylic acid group, proving the dimeric structure of DHA, since the COOH group works both as a donor and acceptor group [43]. In the T-POP3 spectrum, the carboxylic C=O elongation band showed a bathochromic shift from 1717 cm^−1^ to 1653 cm^−1^ possibly due to hydrogen bond formation. The occurrence of these non-covalent interactions and the possible deprotonation of the carboxyl groups due to the presence of basic amine groups in the vicinity allowed explaining the appearance of all O–H and N–H elongations at the same spectral region (3393 cm^−1^).

Overall, the three polymers differ only in the selected dialdehyde and the functional groups present therein; however, this monomer change causes minimal spectral differences, which essentially arise in the fingerprint spectrum region, particularly between 900 and 1250 cm^−1^. Additionally, T-POP3 differs slightly in the region relative to functional groups, due to the presence of carboxyl groups in its structure.

#### 3.1.3. Thermogravimetric Analysis (TGA)

**Figure 3:**
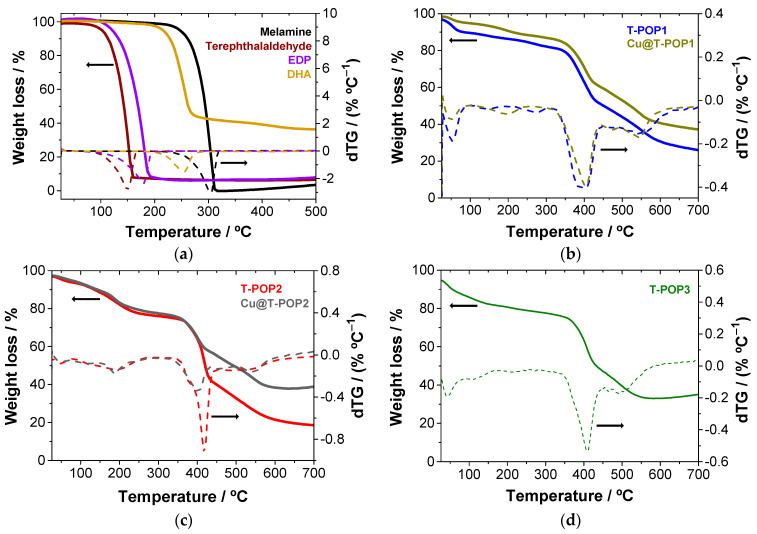
Thermograms (solid line) and dTGs (dotted line) of (**a**) melamine, terephthalaldehyde, 4-ethyl-2,6-diformylphenol (EDP), and 3,5-diformyl-4-hydroxybenzoic acid (DHA); (**b**) T-POP1 and Cu@T-POP1; (**c**) T-POP2 and Cu@T-POP2; and (**d**) T-POP3. The arrows indicate the corresponding ordinate axis.

The thermal stability of monomers and polymers was determined by thermogravimetric analysis (TGA), as shown in Figure 3. The monomers (Figure 3a) present a similar thermal profile with a single degradation step, melamine being the most thermally stable, with a maximum degradation temperature of 306 °C. Concerning dialdehydes, both functionalized dialdehydes show superior thermal stability compared to terephthalaldehyde (149 °C), possibly due to intramolecular hydrogen interactions or intermolecular packaging of aldehyde molecules through hydrogen bonds. Intermolecular association is more likely to occur in the 3,5-diformyl-4-hydroxybenzoic acid due to the formation of dimers, as suggested by FTIR-ATR, which also explains its thermal degradation at 257 °C compared to 181 °C, for 4-ethyl-2,6-diformylphenol.

Degradation curves for polymers (Figure 3b–d) appear practically superimposable, as T-POPs present identical structures with the same type of intra or intermolecular interactions. The event of greater mass loss begins at T > 300 °C and is centered in the range of 402–417 °C. This thermal degradation can be assigned to melamine degradation. Such thermal behavior arises due to the high robustness of the C–N interactions formed between the dialdehyde and melamine and the packing interactions between polymeric layers [44]. Consequently, after Cu(II) complexation with T-POP1 and T-POP2, no significant changes were observed in polymeric structures.

#### 3.1.4. Potentiometric Titration of T-POP3

**Figure 4:**
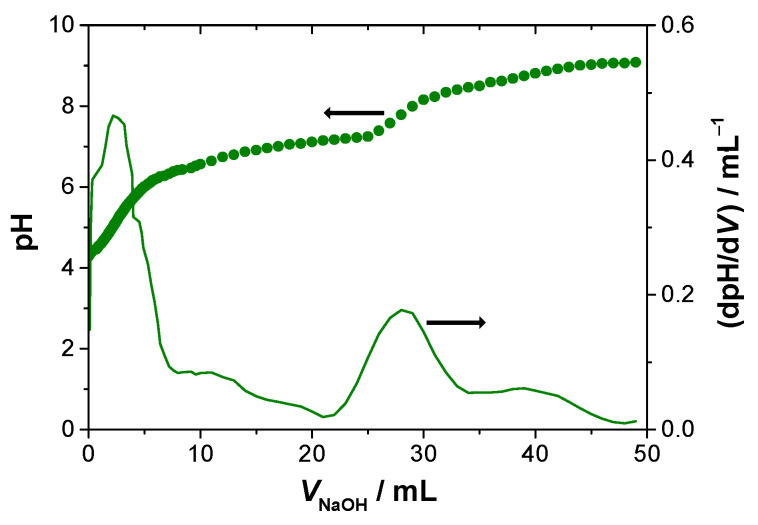
Titration curve (dots) and respective derivative (solid line) of a suspension of T-POP3 (15.2 mg) with a NaOH solution 0.584 mM. The arrows indicate the corresponding ordinate axis.

Potentiometry was used to quantify the carboxylated dialdehyde incorporated in T-POP3. The titration curve (Figure 4) shows two equivalence points, confirming the presence of carboxyl groups in T-POP3: the first neutralization, at pH = 5.2, is due to the deprotonation of carboxyl groups in native form ($R-COOH+{OH}^{-}⇆R-COO^{-}+H_{2}O$) [45], with a p*K*_a_ of 4.7; and the second one, at pH = 7.8, can be justified by the deprotonation of secondary ammonium cations (${R_{2}NH_{2}}^{+}+{OH}^{-}⇆R_{2}NH+H_{2}O$), with a p*K*_a_ of 6.9. The former allows obtaining a 1.0% mass percentage of free carboxylic groups. A total dialdehyde amount incorporated in T-POP3 of 20.9% (by weight) was estimated from the second point; however, most of the carboxyl groups (18.9%) already appear in a deprotonated form after T-POP3 synthesis, namely in a salt form (${R_{2}NH_{2}}^{+}$ ${COO}^{-}$).

#### 3.1.5. Surface area and Porosimetry

**Figure 5:**
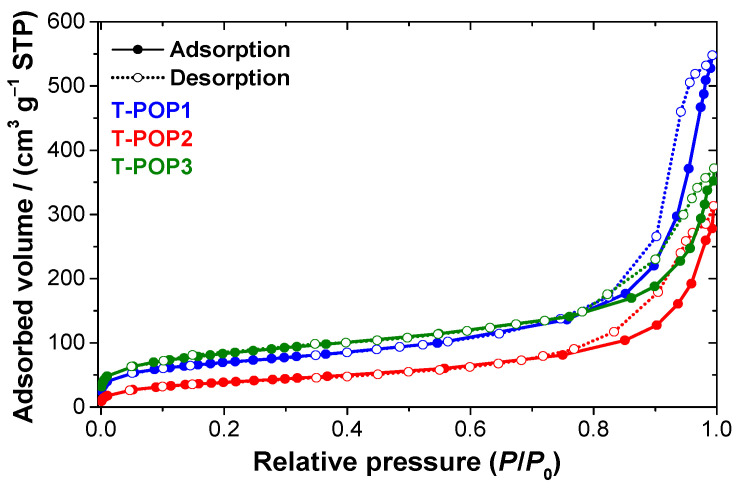
N_2_ adsorption–desorption isotherms of T-POP1, T-POP2, and T-POP3.

The surface and permanent porosity properties of T-POPs were determined by nitrogen sorption (N_2_) at 77 K. Type II gas sorption isotherms were obtained for the three POPs (Figure 5), suggesting mesoporous structures [40,46]. Furthermore, H1-type hysteresis was also observed at high pressures [47].

The parameters obtained by N_2_ sorption, DLS, and ζ-potential analysis are described in Table 1. For the average pore diameter (*d*_p_), the tendency *d*_p_ (T-POP3) < *d*_p_ (T-POP2) < *d*_p_ (T-POP1) was observed, and the smaller pores of T-POP2 and T-POP3 may be related to the structure of the selected starting aldehyde, since dialdehydes EDP and DHA, when compared to terephthalaldehyde (T-POP1), present more functional groups, and its reactive formyl groups appear closer to each other. Additionally, the tightest pore of T-POP3 can also result from the interaction between carboxylic acid groups and adjacent amine groups to form a ${R_{2}NH_{2}}^{+}$ ${COO}^{-}$ salt.

Polymers show high surface areas (*S*_BET_), the surface area for T-POP2 (139.2 m^2^ g^−1^) being lower than those obtained for T-POP1 (239.6 m^2^ g^−1^) and T-POP3 (287.4 m^2^ g^−1^). These results suggest that the polymers with some flexibility at the alkyl secondary amine groups show properties more comparable to HCPs than PIMs [37].

According to data in Table 1, T-POP1 and T-POP2 present a similar particle size, between 110 and 115 nm; T-POP3 particles are of the largest dimension ((225 ± 7) nm) and have the highest PDI (polydispersity index), although all particles can be considered monodisperse [48]. Considering the values obtained for the ζ-potential, the polymers show a high positively charged surface (Table 1), which may result from the protonation of some nitrogen atoms of the triazine rings at pH < 7 [49]. As the ζ-potential values are sufficiently positive, the formation of stable suspensions in water and the favoring of repulsive interactions between the particles would be expected; however, it did not happen using T-POP3 [48]. It can be explained by the sharp decrease in ζ-potential for T-POP3 ((69 ± 3) mV) compared to the values obtained for T-POP1 ((154 ± 9) mV) and T-POP2 ((139 ± 9) mV), resulting from the deprotonation of carboxyl groups, which may favor electrostatic interactions between oppositely charged moieties, leading to larger particles and aggregation.

#### 3.1.6. Scanning Electron Microscopy (SEM)

**Figure 6:**
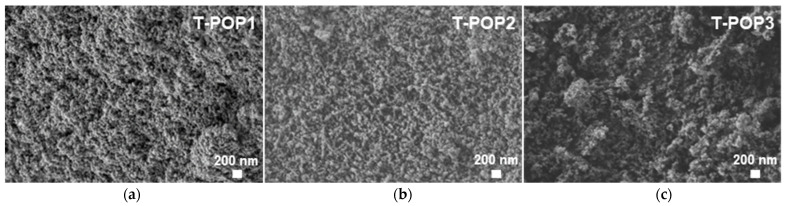

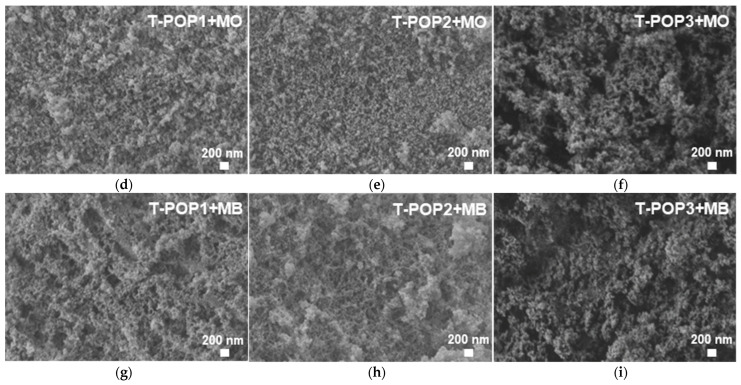
SEM micrographs of neat (**a**) T-POP1, (**b**) T-POP2, and (**c**) T-POP3; after adsorption of methyl orange (MO): (**d**) T-POP1+MO, (**e**) T-POP2+MO, and (**f**) T-POP3+MO; and after sorption of methylene blue (MB): (**g**) T-POP1+MB; (**h**) T-POP2+MB, and (**i**) T-POP3+MB. Magnification 20,000×.

Figure 6 shows SEM images for T-POPs. The surface morphology of T-POP1 and T-POP2 appear spherical, homogeneous, and diffuse, as well as with some roughness, characteristic of porous materials. The monodispersed-sized particle centered at ca. 100 nm and the mesoporosity for T-POP1 and T-POP2 are also visible in the micrographs, confirming the results obtained by N_2_ sorption and DLS. Additionally, the significantly lower *S*_BET_ estimated for T-POP2 is also related to its lower roughness and larger average particle size compared to T-POP1 (Figure 6a,b). On the other hand, the T-POP3 micrograph (Figure 6c) presents spherical, larger, and more dispersed aggregates compared to T-POP1 and T-POP2, as evidenced by DLS measurements. Furthermore, the T-POP3 surface is also more heterogeneous and rougher than that of T-POP1 and T-POP2, also suggesting the porous nature of the polymer.

### 3.2. Dye Adsorption

#### 3.2.1. Methyl Orange (MO) Adsorption

Dyes are a class of organic contaminants with high molecular weight, and most of them are priority pollutants occurring in the wastewater of food, pharmaceutical, textile, paint, plastic, and paper industries [50]. One of them, frequently used as a model organic pollutant to evaluate the adsorption capacity of polymers, is methyl orange (MO), an anionic, water-soluble azo dye, whose diameter ranges between 6 and 8 nm [51]. It can function as a colorant agent in many industries and as a weak acid of p*K*_a_ = 3.5, having a red-to-yellow color change at pH between 3.0 and 4.4 [52].

**Figure 7:**
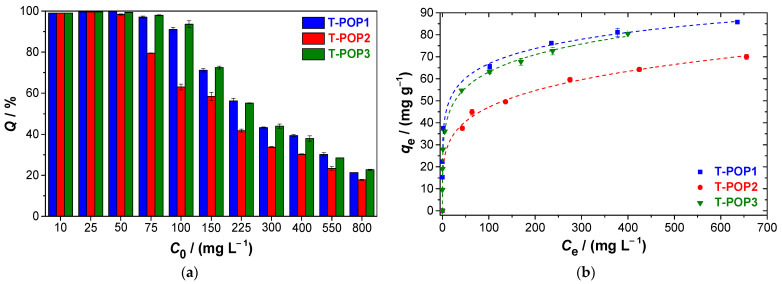
Removal of MO from water: (**a**) effect of initial concentration on adsorption efficiencies and (**b**) adsorption isotherms by different polymers (25 °C). The dashed curves represent the fitting of different models to the experimental data: Hill equation (T-POP1 and T-POP3) and Freundlich equation (T-POP2).

When dissolved in ultrapure water, MO occurs in the basic form, giving it an intense yellow color, which makes the detection possible by UV spectroscopy at 463 nm due to the π → π* transition of the azo group [53]. Figure 7a represents the effect of initial concentration on dye removal efficiency using T-POPs. It can be noticed that sorption capacities decrease with the increase in dye concentration, due to polymer saturation and the increase in dye–dye stacking instead of dye–polymer interaction. As a result, the highest sorption efficiencies are observed starting from 10 mg L^−1^ and 25 mg L^−1^ dye aqueous solutions, since the final content was below the detection limit (0.1 mg L^−1^), corresponding to efficiencies >99.0 and >99.6%, respectively.

Figure 7b depicts representative isotherms for MO adsorption into different T-POPs and the sorption model which best fits each system among the Langmuir, Freundlich, and Hill equations. The fitting parameters of each isotherm model are summarized in Table 2. For T-POP1 and T-POP3, the Hill isotherm model was the best fit for the experimental data, indicating negative cooperative processes, since $n_{H}$ coefficients are below 1 [32,33]. Through this model, the maximum adsorption capacities of $q_{S_{H}}$ = (233 ± 65) mg g^−1^ and (266 ± 72) mg g^−1^ were obtained for MO adsorption on T-POP1 and T-POP3, respectively. On the other hand, the Freundlich equation was the one with a smaller AIC value for MO adsorption on T-POP2, suggesting a chemisorption-controlled process, because ($1/n_{F}$) is less than 1 [31]. However, despite the high potential of the three polymers for removing MO from aqueous media, the interaction adsorbate—T-POP2 is generally more limited. It results from the more compact structure of T-POP2, having a smaller surface area and pore volume accessible to analytes and providing a smaller contact region, which will hinder the adsorption and activation of analytes at the interface. These properties of T-POP2 explain its saturation for lower amounts of dye adsorbed, as shown in Figure 7a,b.

**Figure 8:**
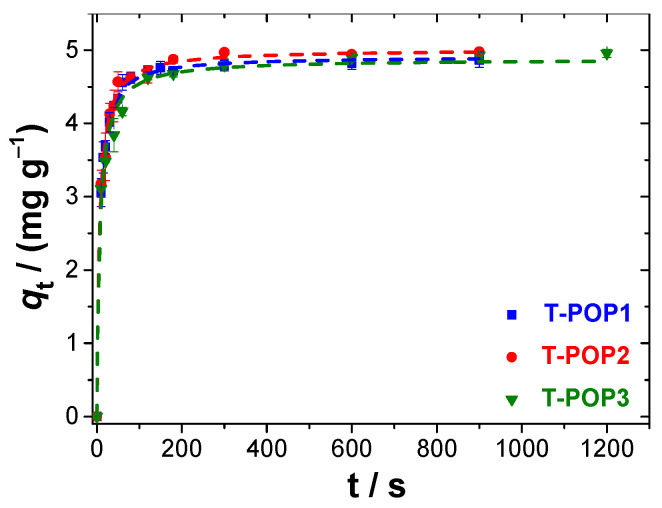
MO adsorption kinetics on different polymers at 25 °C, starting from a 10 mg L^−1^ dye solution. The dashed lines represent the fit of the pseudo-second-order equation.

The construction of the kinetic profiles, together with the sorption isotherms, allows a deeper understanding of the adsorption processes and mechanism. The experimental kinetic data for MO adsorption on T-POPs are shown in Figure 8, and the fitting parameters of the pseudo-first-order and pseudo-second-order models are reported in Table 3. For all materials, the kinetic data follow predominantly the pseudo-second-order equation, which suggests a chemisorption mechanism, possibly due to hydrogen bond formation [34,35]. The high-affinity polymer–dye can also be justified by the occurrence of π–π and electrostatic interactions. From Figure 8, it can also be concluded that polymers remove the dye from water not only efficiently but also quickly (*C*_0_ = 10 mg L^−1^), since MO content becomes below the detection limit in just 15 min for T-POP1 and T-POP2, and 20 min for T-POP3 (achieving efficiencies > 99%). The slight delay of the adsorption process with T-POP3 may be due to its less positive ζ-potential, resulting from the presence of carboxylate groups in its structure.

#### 3.2.2. Methylene Blue (MB) Adsorption

Methylene blue (MB) is a thiazine-type cationic organic dye used for numerous applications such as, for example, in insecticides and microbial agents, dyes in the textile industry, or as an antidote against cyanide intoxication [54,55]. However, MB has harmful effects when disposed of in the environment due to its color, toxicity, high stability, and consequent low biodegradability [56]. Like MO, MB is also considered a standard dye in water decontamination studies, due to the ease of synthesis and quantification by UV spectroscopy, since an involvement of transitions of the type n → π* and π → π* is responsible for the absorbance maximum at 663 nm [57].

**Figure 9:**
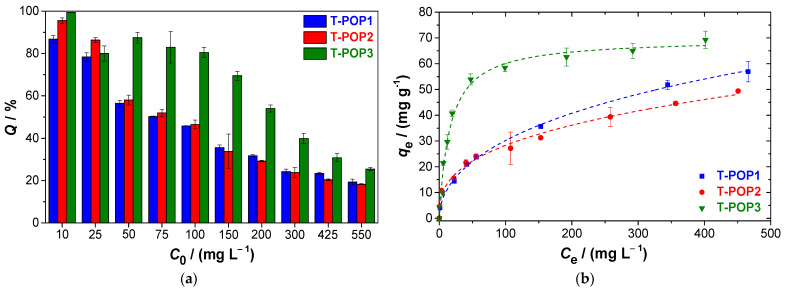
Removal of MB from water: (**a**) effect of initial concentration on adsorption efficiencies and (**b**) adsorption isotherms by different polymers (25 °C). The dashed curves represent the fitting of different models to the experimental data: Hill (T-POP1), Freundlich (T-POP2), and Langmuir (T-POP3) equations.

The efficiencies of MB removal by T-POPs as a function of the initial dye concentration are shown in Figure 9a. It seems clear that the decreasing tendency observed with the increase in dye content may be a result of both the polymeric surface filling closer to the maximum adsorption capacity and the increasing predominance of interactions between dye molecules (with self-aggregation of MB into dimers and eventually tetramers) [58], decreasing its availability for binding with the polymer. Additionally, the presence of functional groups, besides amine groups, in T-POPs (e.g., hydroxyl and carboxyl groups), resulting from pre-synthetic modification of the monomers, leads to an increase in the interaction with the cationic dye MB. Especially with T-POP3, as it has a lower ζ-potential value and greater surface area.

To understand the adsorption mechanism, MB adsorption isotherms were performed using the T-POP adsorbents. The curves fitted by the model that best represents the experimental results are shown in Figure 9b, while the fitting parameters of the models evaluated are described in Table 4. It is noteworthy that the adsorption models of the different polymers differ from one another: Hill isotherm better describes the data for T-POP1, indicating a negative cooperativity in the linkage ($n_{H}<1$) [33]; the Freundlich model represents the adsorption into T-POP2, suggesting a chemisorption mechanism ($\frac{1}{n_{F}}<1$) [31]; and the Langmuir equation was the one that best fitted the sorption data using T-POP3, describing more adequately chemisorption behaviors. Through the fit of the Langmuir equation for MB adsorption by T-POP3, a standard Gibbs energy of adsorption of ${∆G}^{0}$ = (−24.5 ± 0.3) kJ mol^−1^ was calculated, suggesting the homogeneity of the porous surface of T-POP3, with a spontaneous monolayer dye adsorption occurrence [31,59].

According to the best fit for each system, maximum adsorption capacities of (240 ± 137) mg g^−1^ and (70 ± 2) mg g^−1^ were obtained for T-POP1 and T-POP3, respectively. Nevertheless, in the range of concentrations studied (≤550 mg L^−1^), T-POP3 presents greater MB adsorption capacities. On the other hand, T-POP2, when compared to T-POP1, shows superior performance at lower concentrations, but for higher concentrations, MB removal using T-POP1 becomes better, achieving a greater maximum adsorption capacity at the equilibrium plateau (as seen in Figure 9), which may be due to the more disadvantageous morphological and surface properties of T-POP2, namely lower specific surface area and pore volume.

**Figure 10:**
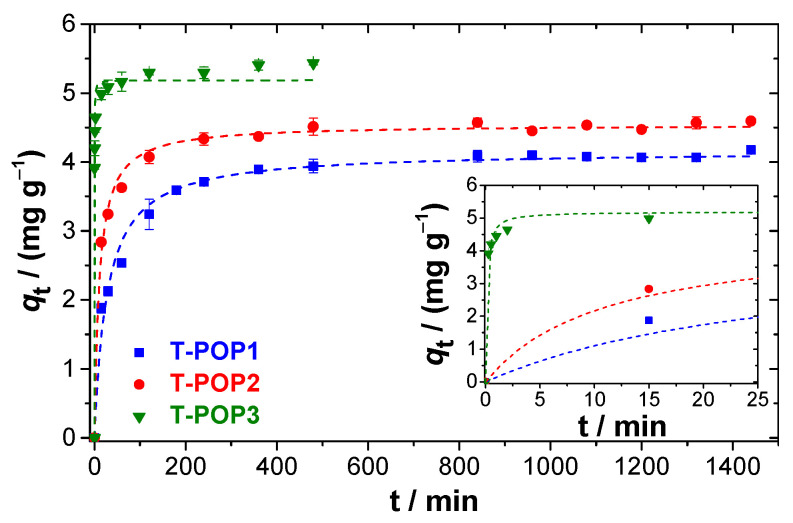
MB adsorption kinetics on different polymers at 25 °C, starting from a 10 mg L^−1^ dye solution. The dashed lines represent the fit of the pseudo-second-order equation and the graph in the bottom right corner corresponds to a magnification between 0–25 min.

To complement the adsorption mechanism studies, the adsorption capacity was followed as a function of incubation time, starting from a 10 mg L^−1^ dye solution (Figure 10). The pseudo-second-order kinetic law equation best fits the experimental data for the three T-POPs (Table 5), suggesting an adsorption process controlled by chemisorption, which may be explained by the establishment of hydrogen bonds between the dye and the surface atoms of the polymers. Although the second-order kinetic equation is predominant, physisorption processes, namely by electrostatic interactions or π-π stacking, are not excluded by this model. For example, π-π non-covalent interactions can occur between aromatic rings, and they may be easily represented by second-order kinetics.

In Figure 10, it was also verified that the equilibrium level for the MB adsorption using T-POP1 and T-POP2 started to be established after ca. 8 h of incubation (480 min), at which the removal efficiencies were around 80% and 90%, respectively. On the other hand, with T-POP3, the adsorption was significantly faster, and the equilibrium level started in less than 15 min, achieving at this time an extent close to 92%. After 8 h, the MB removal by T-POP3 was practically complete, with efficiencies close to 99%.

#### 3.2.3. Methyl Orange (MO) and Methylene Blue (MB) Adsorption

Simultaneous adsorption experiments of both MO and MB using the T-POPs were performed starting from aqueous solutions of 10 mg L^−1^ of each dye. However, a partial neutralization of the two adsorbates was observed over time, leading to a concentration decrease to 7 mg L^−1^ of each dye in the absence of a polymer after incubation conditions. This final value was assumed to be the respective *C*_0_. In turn, for initial concentrations of each dye higher than 10 mg L^−1^, a greenish precipitate formed in the solution, making simultaneous adsorption tests starting from more concentrated solutions impossible to carry out.

**Figure 11:**
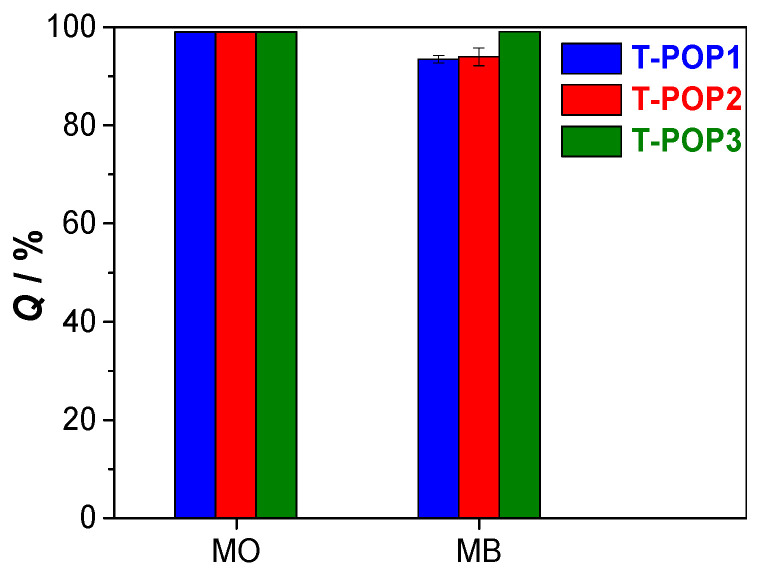
Efficiencies for simultaneous removal of MO and MB by T-POPs, at 25 °C, starting from a solution of nominal concentration of 10 mg L^−1^ and real concentration of 7 mg L^−1^ (values for each dye).

From Figure 11, it can be concluded that T-POPs showed an excellent capacity for the simultaneous removal of both dyes, making an initially greenish solution practically colorless. While T-POP1 and T-POP2 removed MO to a content below the detection limit (efficacy > 99%) and MB with an efficiency of 94%, T-POP3 made the presence of both dyes undetectable after 24 h of incubation, showing greater potential for the simultaneous removal of the studied dyes from water samples.

Overall, dye adsorption causes a decrease in the degree of compaction of the polymeric structure, the appearance of aggregates on the polymer surface, and an increase in the size of the cavities, since the penetration of adsorbates and their diffusion along the pores leads to intermolecular spacing (as observed in the SEM images of Figure 6d–i).

### 3.3. Catalytic Performance in Henry Reaction

Henry reactions or nitro-aldol condensation reactions are commonly base-catalyzed and promote C–C bond formation between nitroalkanes and carbonyl compounds. When the starting nitroalkane has an acidic proton (in the α-position), it can be easily removed in the presence of a basic catalyst, yielding a nucleophilic intermediate (nitronate anion) that will attack the carbonyl carbon of the aldehyde or ketone to form a nitroalcohol. If a primary nitroalkane or nitromethane are selected as reactants, acidic protons will still remain in the nitroalcohol after the addition reaction, making it possible for the water elimination to give rise to nitroalkenes, which correspond to the major secondary product of the Henry reactions under the studied conditions. Thus, a small amount of base and low to moderate temperatures must be used in order to achieve good selectivity for the β-nitroalcohol compounds [60,61]. Typically, an excess of the nitroalkane is added in this equilibrium reaction to ensure the formation of the nitroalcohols in good yields, since they are important building blocks and precursors of a wide variety of other longer-chain compounds, endowed with functional groups essential in chemical synthesis and with biological relevance [62,63].

#### 3.3.1. Comparison between Heterogeneous Catalysts: T-POPs and Cu@T-POPs

**Scheme 1:**
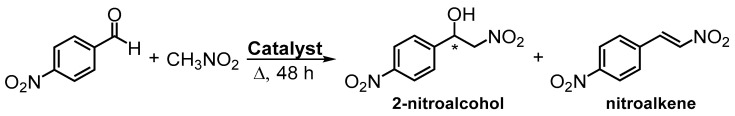
Henry reaction between 4-nitrobenzaldehyde and nitromethane in the presence of a catalyst.

Classical methods for promoting Henry reactions involve catalytic amounts of soluble bases [64]. Alternatively, the development of efficient heterogeneous catalysts, including POFs, is important to overcome the disadvantages of homogeneous catalysis and to make the Henry reactions ecologically more sustainable. In this regard, the synthesized T-POP1 and T-POP2 were tested in the Henry reaction between 4-nitrobenzaldehyde and nitromethane (Scheme 1), as they are polymers with more basic characteristics.

Initially, some reaction parameters were optimized, including solvent, reaction time, and amount of catalyst. It was observed that the best results were obtained using 20 mg of catalyst, a reaction time of 48 h, and nitromethane as solvent and reagent.

Due to the richness in basic amine groups, T-POP1 was the first to be evaluated in the Henry reaction, and the nitroalcohol was obtained in 69% yield at 40 °C (Table 6, entry 3). The consequent rise in temperature to 60 °C caused a significant increase in conversion, to 94%, without affecting the selectivity for 2-nitroalcohol (99%) (Table 6, entry 4). Alternatively, T-POP2 was also used as a heterogeneous basic organocatalyst (Table 6, entries 7 and 8); however, it presented a lower catalytic performance compared to T-POP1, possibly explained by its smaller average pore size, specific pore volume, and surface area.

The abundance of chelating groups in the structure of T-POP1 (NH_2_) and T-POP2 (NH_2_ and OH) enables the formation of strong nitrogen–metal and oxygen–metal interactions, so they can function as starting material for the anchoring of metallic complexes in its structure, in order to obtain organometallic heterogeneous catalysts. This is valuable because transition metal-catalyzed Henry reactions proved to be efficient for obtaining pure nitroalcohols under moderate reaction conditions and with excellent control in selectivity and catalytic activity. Among the diverse transition metals, copper catalysts are advantageous because of their less toxic and more economical nature [63]. Generically, the mechanism of this catalytic reaction involves a transition metal complex as a weak Lewis acid to which the oxygen atoms of the nitroalkane and the aldehyde will simultaneously coordinate, leading to the approximation of the reactive species and the activation of the carbonyl compound under the mild acidic conditions; and moderately basic counter anions, such as acetate ligands, which will deprotonate the α-carbon atom of the nitroalkane to generate the nitronate anion and trigger the reaction [8,65].

Cu@T-POP catalysts were prepared by post-synthetic metalation of T-POPs using Cu(OAc)_2_∙H_2_O in methanol at 60 °C. At these conditions, (217 ± 10) and (178 ± 10) mg of Cu(II) were retained per gram of T-POP1 and T-POP2, respectively, resulting in Cu@T-POP1 and Cu@T-POP2. Using both copper catalysts, identical catalytic activity in the Henry reaction was observed at 40 °C and 60 °C. At 40 °C, excellent conversions of 97% and high selectivities for the 2-nitroalcohol (93–94%) were obtained (Table 6, entries 5 and 9); however, the increase in temperature to 60 °C caused a 2-nitroalcohol selectivity decrease (Table 6, entries 6 and 10), due to the favoring of water elimination reaction with heating.

The maximum incorporation of Cu(II) was (217 ± 10) mg g^−1^ for T-POP1, which is equivalent to ca. 2.6 mg of Cu(II) and 8.3 mg of Cu(OAc)_2_∙H_2_O per 20.0 mg of Cu@T-POP1, the amount used in the Henry reaction catalysis. Therefore, the mentioned content of Cu(OAc)_2_∙H_2_O complex (8.3 mg) was used to promote the nitro-aldol condensation (Table 6, entry 2). Only 10% conversion was obtained in these conditions. This result can be explained by considering the dimeric structure of copper(II) acetate monohydrate in solid state. Upon reaction with T-POPs, the copper complex adopts a monomeric form, and an increase in the accessibility of the species to the metallic center occurs, explaining the higher catalytic activity of Cu@T-POPs [8].

In summary, the best conditions found to promote the nitro-aldol condensation between 4-nitrobenzaldehyde and an excess of nitromethane were a 48 h reaction at 40 °C, using Cu@T-POP1 or Cu@T-POP2 as the catalyst. It is also possible to conclude that Cu@T-POP1 and Cu@T-POP2 led to higher conversions than T-POPs, even at lower temperatures, without a significant decrease in the selectivities, which were 93–94% with Cu@T-POPs under optimized conditions, vs. 98–99% for T-POPs (at 60 °C). Compared with other heterogeneous catalysts in the literature, such as zeolites [66], POPs [67,68], CTFs [8], MOFs [8,69], and chitosan [70] or cellulose-based [71] materials, T-POPs, and especially the Cu@T-POPs, showed comparable Henry reaction efficiencies. In certain situations, Cu@T-POPs even showed better results than MOFs and other copper-based materials, taking advantage of achieving high performances in the absence of solvent and at more moderate temperatures [8,70]. In addition, the polymers synthesized in this work represent a breakthrough for science in the search for efficient materials, due to their dual purpose and low cost, especially for the T-POP1 skeleton.

#### 3.3.2. Recyclability Studies

**Figure 12:**
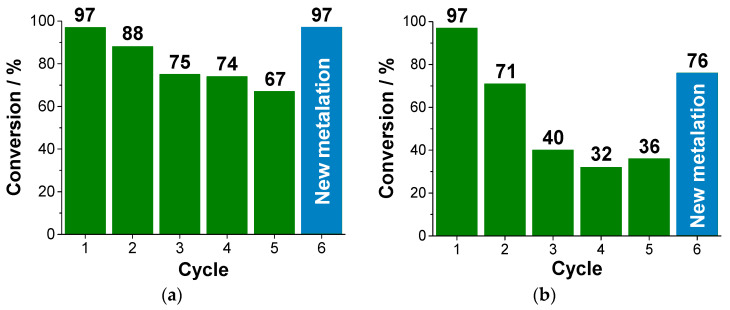
Effect of catalyst reuse cycles on conversion efficiency: (**a**) Cu@T-POP1 and (**b**) Cu@T-POP2.

The catalysts with the best catalytic performance (Cu@T-POP1 and Cu@T-POP2) were recovered by centrifugation followed by liquid phase decantation and were subsequently used in six successive catalytic cycles under the optimized conditions (48 h at 40 °C), as shown in Figure 12. There was a decrease in the catalytic activity along the reuses of Cu@T-POP1, mainly due to copper leaching, since the conversion recovered the initial value after the new metalation of the remaining polymer (cycle 6) (Figure 12a). In comparison, Cu@T-POP2 showed a catalytic performance identical to Cu@T-POP1 in the first use, followed by a sharper catalytic activity decrease in reuse, which was only partially recovered (to 76%) after re-metalation (Figure 12b). This observation may be explained by the relevant catalyst amount decrease (due to unavoidable losses during recovery and washing procedures), copper leaching, and poor characteristics of the starting polymer, T-POP2, namely smaller specific surface area, pore size, and pore volume. In general, the nitroalcohol selectivities remained unaffected in all six cycles (between 91 and 100%).

#### 3.3.3. Expansion of the Substrate Scope

Proving to be the most efficient catalyst gathering the catalytic activity and reuse, using 4-nitrobenzaldehyde as the substrate, Cu@T-POP1 was used to investigate the scope of the reaction using several aromatic aldehydes (Table 7). The results presented in Table 7 show that conversions between 4 and 97% and excellent selectivities (>83%) are observed under the optimized reaction conditions.

From the results, it was possible to verify that the substituent group of the aromatic aldehydes and its position relative to the formyl group had a pronounced effect on the Henry reaction conversions. Generally, aromatic aldehydes with electron-withdrawing groups led to higher catalytic activities when compared to those obtained with substrates with electron-donating groups. For example, a higher conversion of 97% was afforded using 4-nitrobenzaldehyde when compared to that observed using 4-methylbenzaldehyde (36%), as shown in Table 7, entries 3 and 11. This results from the fact that electron-withdrawing groups increase the electrophilicity of the carbonyl carbon and, consequently, its susceptibility to undergo the nucleophilic attack of the nitronate anion formed by deprotonation of nitromethane [72,73,74]. Regarding the substituent position, a higher stereochemical hindrance occurs when it is in the ortho position to the formyl group, hampering the approach and the attack of the nitronate anion (nucleophile) on the carbonyl carbon. For this reason, higher conversions were obtained when aldehydes with substituent groups located in the para position of the aromatic ring or 2-naphthalaldehyde (compared to 1-naphthalaldehyde) were used as substrates [75].

The increase in the reactions’ temperature from 40 °C to 60 °C was also evaluated, with the aim of increasing the extension of the reactions (particularly when using aromatic aldehydes with an electron-donating group); however, a significant loss in selectivity was again observed.

## 4. Conclusions

By polycondensation reaction between melamine and a dialdehyde (non-functionalized or functionalized), in the molar ratio 2:3, and through a conventional solvothermal method, three reticulated, thermally stable, and nitrogen-rich colloidal-sized POPs (T-POP1, T-POP2, and T-POP3) were obtained. The use of aldehydes with different functionalities allowed the modulation of physical properties, which proved to be crucial in the materials’ performance. It was observed that the *S*_BET_ for T-POP2 was clearly lower than those obtained for T-POP1 and T-POP3, and that T-POP3 presented smaller mesopores (8.0 nm) compared to T-POP1 (14.2 nm) and T-POP2 (13.9 nm), given the greater proximity between formyl groups and greater functionalization of the starting aldehyde (also valid for T-POP2), as well as due to the presence of carboxyl groups, mostly deprotonated and possibly in salt form. As a result of the higher density of opposite charges, T-POP3 also showed a more heterogeneous and aggregated surface, as well as a surface with lower positive charge density.

In adsorption studies, all POPs proved to be excellent adsorbents of methyl orange anionic dye, removing it from 10 mg L^−1^ aqueous solutions with efficiencies >99% in only 15–20 min. In methylene blue cationic dye removal from water (10 mg L^−1^), efficiencies of (87 ± 2)%, (96 ± 1)%, and ca. 99.4% for the T-POP1, T-POP2, and T-POP3 were achieved, respectively. For this dye removal, a performance increase according to the decrease in positive ζ-potentials were observed, given how the efficiencies are particularly higher with T-POP3, possibly due to favorable interactions via deprotonation of its carboxyl groups, which also contributed to a significantly faster adsorption process on this polymer. The maximum adsorption capacities of methyl orange, equal to (233 ± 65) mg g^−1^, (152 ± 117) mg g^−1^ and (266 ± 72) mg g^−1^, respectively, for T-POP1, T-POP2, and T-POP3 (calculated from the Hill isotherm fit), showed a significantly lower value for T-POP2 due to its smaller *S*_BET_. The same conclusion was obtained when adsorbing methylene blue, and, in this case, T-POP3 showed the highest adsorption capacities in the range of concentrations under study. The pseudo-second-order kinetic best fitting for all situations also confirmed the occurrence of chemisorption.

In the Henry reaction catalysis, the best results were obtained using Cu@T-POPs as catalysts, and nitromethane as the solvent and reagent, at 40 °C for 48h. Good conversions (up to 97%) and selectivities for nitroalcohols (up to 100%) were obtained, especially using aromatic aldehydes with electron-withdrawing groups and substituted in the para position, as the nucleophilic attack of the nitronate anion is facilitated by the electropositivity increase in the carbonyl carbon and by a lower steric hindrance, respectively. The best results in reuse were achieved using Cu@T-POP1 since T-POP1 has a greater surface area, pore diameter, and pore volume when compared to T-POP2. Overall, Cu@T-POP1 was the most efficient catalyst.

In conclusion, dual-purpose POPs have been successfully synthesized. Their applicability in heterogeneous catalysis brings an overview to carry out synthetic processes based on Henry reactions greener and more efficiently, while its application in water treatment has shown high efficiency, quickness, and versatility for the removal of dyes with different ionic charges. This suggests a relevant contribution to the development of methods and low-cost materials for, e.g., textile effluent remediation. This work is also paving the way for the development of multifunctional materials.

## Figures and Tables

**Table 1:** Parameters obtained by N_2_ sorption, DLS, and ζ-potential analysis.

Material	*S*_BET_/m^2^ g^−1^	Pore Diameter/nm	Pore Volume/cm^3^ g^−1^	Particle Size/nm	PDI	ζ-Potential/mV
T-POP1	239.6	14.2	0.85	109.8 ± 0.3	0.11 ± 0.01	154 ± 9
T-POP2	139.2	13.9	0.48	114.8 ± 0.2	0.12 ± 0.02	139 ± 9
T-POP3	287.4	8.0	0.58	225 ± 7	0.30 ± 0.03	69 ± 3

**Table 2:** Fitting parameters of different models of isotherms for MO adsorption.

Model	Parameters	Adsorbent		
		T-POP1	T-POP2	T-POP3
Langmuir $q_{e}=\frac{q_{m}K_{L}C_{e}}{1+K_{L}C_{e}}$	$q_{m}$/($mg\;g^{-1}$)	78 ± 6	71 ± 3	69 ± 5
	$K_{L}$/($L\;{mg}^{-1}$)	0.5 ± 0.3	0.024 ± 0.004	0.4 ± 0.2
	$R^{2}$	0.8360	0.9303	0.8516
	AIC	18.0	9.1	20.9
Freundlich $q_{e}={K_{F}C_{e}}^{1/n_{F}}$	$K_{F}$/(${mg}^{1-1/n_{F}}g^{-1}L^{1/n_{F}}$)	31.4 ± 0.9	18 ± 1	26.5 ± 0.6
	$1/n_{F}$	0.159 ± 0.005	0.21 ± 0.01	0.186 ± 0.005
	$R^{2}$	0.9970	0.9845	0.9977
	AIC	5.9	5.2	4.6
Hill $q_{e}=\frac{q_{S_{H}}C_{e}^{n_{H}}}{K_{D}+C_{e}^{n_{H}}}$	$q_{S_{H}}$/($mg\;g^{-1}$)	233 ± 65	152 ± 117	266 ± 72
	$K_{D}$/$\left((mg\;L^{-1}{)}^{n_{H}}\right)$	6 ± 2	10 ± 5	9 ± 3
	$n_{H}$	0.20 ± 0.02	0.3 ± 0.1	0.23 ± 0.01
	$R^{2}$	0.9991	0.9963	0.9992
	AIC	4.4	6.5	2.5

**Table 3:** Fitting parameters of different kinetic models for MO adsorption.

Model	Parameters	Adsorbent		
		T-POP1	T-POP2	T-POP3
Pseudo-first-order $q_{t}={q_{e}(1-e}^{-k_{1}t})$	$q_{e}$/($mg\;g^{-1}$)	4.65 ± 0.06	4.7 ± 0.1	4.6 ± 0.1
	$k_{1}$/(${min}^{-1}$)	5.3 ± 0.4	4.9 ± 0.6	4.95 ± 0.01
	$R^{2}$	0.9748	0.9649	0.9374
	AIC	−18.1	−10.9	−6.6
Pseudo-second-order $q_{t}=\frac{k_{2}q_{e}^{2}t}{1+k_{2}q_{e}t}$	$q_{e}$/($mg\;g^{-1}$)	4.91 ± 0.02	5.01 ± 0.05	4.88 ± 0.07
	$k_{2}$/($g\;{mg}^{-1}\;{min}^{-1}$)	1.98 ± 0.08	1.8 ± 0.2	1.687 ± 0.004
	$R^{2}$	0.9982	0.9943	0.9876
	AIC	−35.1	−20.3	−14.4

**Table 4:** Fitting parameters of different models of isotherms for MB adsorption.

Model	Parameters	Adsorbent		
		T-POP1	T-POP2	T-POP3
Langmuir $q_{e}=\frac{q_{m}K_{L}C_{e}}{1+K_{L}C_{e}}$	$q_{m}$/($mg\;g^{-1}$)	67 ± 4	52 ± 5	70 ± 2
	$K_{L}$/($L\;{mg}^{-1}$)	0.010 ± 0.002	0.015 ± 0.005	0.062 ± 0.008
	$R^{2}$	0.9777	0.9028	0.9812
	AIC	9.5	16.2	13.3
Freundlich $q_{e}={K_{F}C_{e}}^{1/n_{F}}$	$K_{F}$/(${mg}^{1-1/n_{F}}\;g^{-1}L^{1/n_{F}}$)	4.0 ± 0.3	5.6 ± 0.5	16 ± 3
	$1/n_{F}$	0.43 ± 0.01	0.35 ± 0.02	0.26 ± 0.04
	$R^{2}$	0.9976	0.9906	0.8973
	AIC	2.7	6.0	20.7
Hill $q_{e}=\frac{q_{S_{H}}C_{e}^{n_{H}}}{K_{D}+C_{e}^{n_{H}}}$	$q_{S_{H}}$/($mg\;g^{-1}$)	240 ± 137	-	67 ± 3
	$K_{D}$/$\left((mg\;L^{-1}{)}^{n_{H}}\right)$	72 ± 32	-	22 ± 9
	$n_{H}$	0.51 ± 0.05	-	1.2 ± 0.2
	$R^{2}$	0.9986	-	0.9855
	AIC	2.7	-	15.3

**Table 5:** Fitting parameters of different kinetic models for MB adsorption.

Model	Parameters	Adsorbent		
		T-POP1	T-POP2	T-POP3
Pseudo-first-order $q_{t}={q_{e}(1-e}^{-k_{1}t})$	$q_{e}$/($mg\;g^{-1}$)	3.9 ± 0.1	4.40 ± 0.08	5.1 ± 0.1
	$k_{1}$/(${min}^{-1}$)	0.023 ± 0.003	0.052 ± 0.006	5.0 ± 0.9
	$R^{2}$	0.9338	0.9546	0.9474
	AIC	−12.4	−13.1	−8.1
Pseudo-second-order $q_{t}=\frac{k_{2}q_{e}^{2}t}{1+k_{2}q_{e}t}$	$q_{e}$/($mg\;g^{-1}$)	4.16 ± 0.06	4.54 ± 0.04	5.19 ± 0.07
	$k_{2}$/($g\;{mg}^{-1}\;{min}^{-1}$)	0.0087 ± 0.0009	0.020 ± 0.002	1.9 ± 0.3
	$R^{2}$	0.9832	0.9926	0.9829
	AIC	−21.3	−24.1	−14.0

**Table 6:** Influence of catalyst type on the Henry reaction between 4-nitrobenzaldehyde and nitromethane.

Entry *	Catalyst	T/°C	Conversion/% ^a^	2-nitroalcohol/% ^a^
1	---	60	2	100
2	Cu(OAc)_2_∙H_2_O	60	10	95
3	T-POP1	40	69	100
4	T-POP1	60	94	99
5	Cu@T-POP1	40	97	93
6	Cu@T-POP1	60	96	82
7	T-POP2	40	51	100
8	T-POP2	60	84	98
9	Cu@T-POP2	40	97	94
10	Cu@T-POP2	60	92	85

^a^ Values determined by ^1^H-NMR spectroscopy. * Aldehyde (0.80 mmol), excess of nitromethane (2 mL), as solvent and reagent, catalyst T-POP/Cu@T-POP (20.0 mg) or Cu(OAc)_2_∙H_2_O (8.3 mg), and 48 h of reaction. Cu@T-POPs were obtained by metalation of T-POPs with Cu(OAc)_2_∙H_2_O in methanol at 60 °C.

**Table 7:** Substrate effect on Henry reactions with nitromethane catalyzed by Cu@T-POP1.

Entry *	Substrate	Conversion/% ^a^		2-nitroalcohol/% ^a^	
		40 °C	60 °C	40 °C	60 °C
1	2-nitrobenzaldehyde	80	92	100	81
2	3-nitrobenzaldehyde	94	91	90	88
3	4-nitrobenzaldehyde	97	96	93	82
4	2-chlorobenzaldehyde	61	93	100	87
5	3-chlorobenzaldehyde	18	45	100	87
6	2-bromobenzaldehyde	47	89	98	93
7	4-bromobenzaldehyde	75	77	91	67
8	Benzaldehyde	59	52	84	78
9	2-methylbenzaldehyde	11	44	100	70
10	3-methylbenzaldehyde	4	24	100	83
11	4-methylbenzaldehyde	36	58	85	66
12	1-naphthaldehyde	20	50	100	66
13	2-naphthaldehyde	57	64	83	53

^a^ Values determined by ^1^H-NMR spectroscopy. * Aldehyde (0.80 mmol), excess of nitromethane (2 mL) as solvent and reagent, catalyst Cu@T-POP1 (20.0 mg), and 48 h of reaction. Cu@T-POP1 was obtained by metalation with Cu(OAc)_2_∙H_2_O (0.80 mmol) in 60 mL of methanol at 60 °C.

## Data Availability

Not applicable.
